# Exploring Burnout, Work Addiction, and Stress-Related Growth among Prehospital Emergency Personnel

**DOI:** 10.3390/bs14090851

**Published:** 2024-09-22

**Authors:** Monica Puticiu, Mihai-Bujor Grecu, Luciana Teodora Rotaru, Mihai Alexandru Butoi, Gabriela Vancu, Mihaela Corlade-Andrei, Diana Cimpoesu, Raluca Mihaela Tat, Adela Golea

**Affiliations:** 1 Department of Emergency, Faculty of Medicine, Vasile Goldis Western University of Arad, 310025 Arad, Romania; puticiu.monica@uvvg.ro; 2 UPU-SMURD “Pius Brînzeu” Emergency Hospital, 300723 Timișoara, Romania; mihai.grecu@urgentatm.ro; 3 Emergency Medicine and First Aid Department, Faculty of Medicine, University of Medicine and Pharmacy, 200349 Craiova, Romania; luciana.rotaru@umfcv.ro (L.T.R.); mihai.butoi@rmu.smurd.ro (M.A.B.); 4 Department of Psychology, Faculty of Educational Sciences, Psychology and Social Assistance, University Aurel Vlaicu Arad, Elena Drăgoi Street, No. 2, 310032 Arad, Romania; gabriela.vancu@uav.ro; 5 Faculty of Medicine, University of Medicine and Pharmacy “Grigore T. Popa”, 700115 Iasi, Romania; 6 Emergency “St. Spiridon” Hospital, 700111 Iasi, Romania; 7 Surgery Department—Emergency Medicine Discipline, University of Medicine and Pharmacy “Iuliu Hațieganu”, 400347 Cluj, Romania; raluca.tat@umfcluj.ro (R.M.T.); adela.golea@umfcluj.ro (A.G.)

**Keywords:** emergency personnel, burnout, work addiction, responsive distress, self-discipline, stress-related growth

## Abstract

Burnout and stress-related issues are significant concerns among medical personnel involved in emergency situations due to the high demands of their work. A cross-sectional descriptive and comparative study was conducted on 266 prehospital emergency personnel across five Romanian counties, comprising 41 physicians, 74 nurses, and 151 paramedics. Data were collected through an online form, including demographic and professional characteristics, and five validated scales. This study revealed moderate (49.3%) to high (25.9%) burnout rates, with 35% showing signs of work addiction. Despite these challenges, the personnel demonstrated high levels of stress-related growth (61.2%), strong self-discipline (74.1%), and low to moderate responsive distress (100%). Physicians exhibited higher work addiction and job satisfaction, whereas paramedics faced higher burnout, self-discipline, and distress levels. Nurses showed lower burnout and self-discipline levels. These findings highlight the prevalence of burnout and work addiction among emergency medical personnel, while also underscoring the presence of protective factors like higher self-discipline, good level of stress-related growth, and low to moderate responsive distress. The distinct differences in experiences among physicians, nurses, and paramedics emphasize the need for tailored strategies to address these issues within each group.

## 1. Introduction

Emergency medicine (EM) is a medical specialty focused on the immediate diagnosis and treatment of illnesses or injuries. EM medical care operates in two modes: prehospital and in-hospital interventions [1]. Our study specifically focuses on prehospital EM personnel.

EM medical care is characterized by heavy workloads, rapid transitions, time pressure situations, uncertainty, often danger, and a necessity for high efficiency [2]. Ambulance EM teams are the first to attend the patients, requiring them to make quick and often life-saving decisions outside the hospital environment, with limited resources and under significant time pressure. EM personnel are often exposed to intense stressful situations which can affect not only the quality of the medical services, but also their own health and personal lives [3].

Burnout is a syndrome characterized by three major dimensions: exhaustion, disengagement, and a sense of ineffectiveness regarding one’s work [4]. The term burnout, coined by Herbert Freudenberger, is described as a state of mental and physical exhaustion caused by the challenges of professional life [5]. Occupational burnout among EM personnel is a significant concern due to the highly stressful conditions. This can lead to medical errors, interpersonal conflict, fatigue, employee turnover, increased absenteeism, and mental health problems [2,6,7].

A 2020 meta-analysis [8] shows that 40% of EM physicians exhibit high levels of emotional exhaustion, 41% show high levels of depersonalization, and 35% experience low levels of personal accomplishment, as rated by the Maslach Burnout Inventory. Somville et al. [9] argue that burnout levels among EM physicians have increased in the last decade, with prevalence rates ranging between 43% and 54%, and Colville et al. [10] report similarly high levels of burnout among EM healthcare workers, ranging from 30% to 60%, with a greater risk for physicians than nurses. Liu et al. [2] found a global burnout rate of 33.4% in a sample of 2299 EM personnel (physicians and nurses) from Chengdu, China and a burnout rate of 36.94% for physicians and 31.11% for nurses.

Baier et al. [11] reported burnout rates between 19% and 40% among 1101 German prehospital EM health workers. In Riyadh, 63% of Saudi physicians experienced emotional exhaustion, and 40% reported depersonalization/disengagement [12]. A study of 327 EM physicians in India found a burnout rate of 28.7% [13].

Yao Xiuyu et al. [14] studied 256 EM nurses from several hospitals in Beijing, China, finding that 31.6% experienced severe burnout. Rodriguez-Rey et al. [15] reported a 57% burnout rate and 72.8% low perceived well-being among Spanish pediatric critical care personnel. In Japan, Morikawa et al. [3] found that among EM physicians, 8.9% reported high burnout, 16.1% experienced severe emotional exhaustion, 19.8% had high depersonalization, and 67% scored low on personal accomplishment.

A previous study on Romanian EM personnel [16] involving 184 participants (physicians, nurses, carers, paramedics, stretcher-bearers, registrars) found that 30.2% are at risk of burnout, which is linked to low work satisfaction and factors like social support and feedback. EM personnel are exposed to intense stress due to their work, increasing the risk of emotional distress, work–life imbalance, and burnout. Burnout rates in EM physicians are higher than in general physicians and significantly exceed those in the general population [8,10,17].

Work addiction, initially described by Oates as an uncontrollable need to work, is now defined as an irresistible drive to work excessively and/or compulsively [18]. It involves working beyond job requirements and becoming obsessively engaged in work. Work addiction is a behavioral addiction (non-substance) with harmful consequences, distinct from a hard worker who enjoys their work [19]. It is linked to poor health, low well-being, and work–family conflicts [20]. Those addicted to work prioritize their jobs, define their self-worth through work, spend excessive time on work, ignore health issues, and often return to work prematurely after illness. This addiction can cause severe physical and mental health problems, including fatigue, hypertension, insomnia, cardiovascular issues, gastritis, alopecia, anxiety, and depression [21].

Rates of work addiction vary widely among different occupational groups and are due to the use of diverse models. Merchaoui et al. [22] report that rates among physicians range from 8.3% to 30%. A study on 444 French university hospital physicians found that 13% exhibited high work addiction, while 35% showed mild addiction [23]. Rates of work addiction among nurses are similar. Ruiz-Garcia et al. [24] found that 28.3% of a total of 219 Spanish emergency and critical care nurses exhibited scores indicative of work addiction. To our knowledge, no study has been conducted on work addiction among Romanian EM personnel.

Responsive distress is the tendency to experience negative emotions in response to others’ distress, focusing on the observer’s discomfort rather than concern for the suffering person. Unlike empathic concern, which involves care for others, responsive distress centers on one’s own negative feelings. Additionally, blunted reward responsiveness is a key risk factor for depression, particularly under stress. Studies show that individuals with lower reward responsiveness at baseline are more vulnerable to anxiety and depression during stressful events, highlighting the need to address this vulnerability in stress-related interventions [25,26].

Self-discipline, defined by Harrison Gough and measured by the Self-Discipline Scale of the California Psychological Inventory [27], refers to the ability to exercise self-control and prioritize actions over emotions. This trait is crucial for EM personnel, particularly those in the Emergency Mobile Service for Resuscitation and Extrication (SMURD), which operates as a military-like structure within the Inspectorate for Emergency Situations (ISU). Burnout, a significant issue in emergency nursing, is linked to higher turnover rates, traumatic events, shift work, violence, and stress from the COVID-19 pandemic [28]. Building resilience through self-discipline, optimism, and goal-oriented behaviors is essential to combating burnout in this high-stress environment.

Stress is an undeniable reality of human life and almost everyone will experience a stressful situation during their lifetime [29]. Post-traumatic growth or stress-related growth is defined as the experience of useful changes following exposure to a stressful event [30]. Stress-related growth works in three distinct directions: improved social relations, increased trust in personal resources, and enhanced coping abilities. Park et al. [31] argue that stress-related growth has been observed even in extremely stressful situations such as the death of a loved one, severe illness, divorce, or accidents.

Stress is a universal experience that can lead to stress-related growth, manifesting as improved social relationships, enhanced personal resources, and better coping abilities [29,30,31]. During the COVID-19 pandemic, healthcare workers (HCWs), particularly physicians, faced significant trauma but also demonstrated post-traumatic growth (PTG). A study of 691 healthcare workers in Kosovo highlighted that despite mental health challenges, including anxiety, depression, and insomnia, positive changes were noted in areas like relating to others and personal strength [32]. Physicians, who exhibited higher resilience than the general population, were identified as an “at-risk” group due to their exposure to infected environments. Resilience in physicians is crucial, as it not only mitigates anxiety and depression but also fosters PTG. Ensuring adequate social and psychological support, along with time for rest and family, is essential in helping HCWs, especially physicians, manage the mental health impacts of the pandemic and promote long-term PTG [33].

Research on burnout and stress-related issues among Romanian emergency medical (EM) personnel is limited [16]. To the best of our knowledge, no studies have yet explored the relationship between burnout, work addiction, and stress management variables (such as stress-related growth) within this population. Additionally, there has been little investigation into the differences between various categories of EM personnel, including physicians, nurses, and paramedics.

Therefore, our objective was to evaluate burnout, work addiction, distress response, self-discipline, and stress-related growth among EM personnel, while also identifying differences among physicians, nurses, and paramedics. We focus on two main hypotheses. First, we hypothesize that EM personnel exhibit moderate to high levels of burnout and work addiction, while simultaneously demonstrating strong self-discipline, low distress responses, and a significant capacity for stress-related growth. Second, we anticipate identifying significant differences among EM physicians, EM nurses, and paramedics across all relevant variables, given their distinct roles, training backgrounds, and experiences within EM teams.

## 2. Materials and Methods

### 2.1. Participants and Procedure

This cross-sectional descriptive and comparative study of 266 EM personnel included physicians, nurses, and paramedics. A total of 728 individuals were addressed for this study (170 EM physicians, 400 EM nurses, 158 paramedics), from 5 out of the 41 counties from Romania. A total of 266 individuals completed the online form, indicating a 36.5% response rate.

Informed consent was obtained through the online form, which was distributed to prehospital EM personnel from 5 Romanian counties, between December 2023 and February 2024. A total of 266 individuals completed the online form, comprising 41 physicians, 74 nurses, and 151 paramedics.

In Romania, the paramedics are part of ISU, a military structure under the Ministry of Internal Affairs that also operates the firefighting services. Physicians and nurses are employed by the local or regional public hospitals, and team up with paramedics within the prehospital EM mobile teams, part of the SMURD, operating within the ISU regional structures. Paramedics work by rotation within two types of EM teams: the mobile intensive therapy units (1 physician, 1 nurse, and 2 paramedics) and the specialized first-aid units (3 paramedics).

### 2.2. Measures

Participants completed an online form comprised two sections. First section focused on demographic and professional characteristics, including age, gender, field experience, type of EM personnel (physician, nurse, paramedic), health characteristics: smoking status, self-evaluated health status on a five-point scale, acute and chronic medical conditions, sleep duration outside working shifts, stress-related post-work activities, work satisfaction, and subjective well-being. The last two variables were both assessed on a ten-point scale, ranging from 1 (extremely low) to 10 (extremely high).

Section two comprised five scales: The Oldenburg Burnout Inventory (OLBI), The Dutch Work Addiction Scale (DUWAS), Responsive Distress Scale (RD), Self-discipline Scale (SFD) and The Stress-Related Growth Scale (SRGS). First four scales were retrieved from Romanian version of International Personality Item Pool (IPIP) at https://researchcentral.ro (accessed on 25 March 2024). SRGS is part of the Clinical Assessment System (SEC), a licensed set of psychometric instruments developed, adapted, and distributed by RTS Romanian Psychological Testing Services.

OLBI is a 16-item self-reported scale assessing two core dimensions of burnout: disengagement and exhaustion [34]. It is based on the job demands–resources model [35], which states that working conditions can be delimited in two broad categories: demands and resources, the lack of which leads to disengagement. Exhaustion (8 items) refers to feelings of emotional drain and emptiness, fatigue (physical and mental), and the need for rest. Disengagement from work (8 items) refers to the tendency of distancing oneself from work, while manifesting a negative perception about work and even cynical attitudes and behaviors towards it [34]. Items are scored on a 4-point Likert scale, where 1—strongly disagree to 4—strongly agree. Total score can range between 16 and 64, with higher scores indicating higher levels of exhaustion, disengagement, or burnout (cumulative score of the two dimensions). For our sample, reliability for the entire scale was adequate, Cronbach’s Alpha = 0.826, disengagement (0.444), and exhaustion (0.817).

DUWAS-10 is used to assess work addiction and its two dimensions: working excessively (WE) and working compulsively (WC). Each subscale has 5 items with scoring on a four-point Likert scale, 1—never to 4—always. Raw scores are divided per number of items and can range from 1 to 4. Scores above 75th percentile are considered relevant for the construct [36,37]. Reliability for DUWAS-10 was good for our sample, Cronbach’s Alpha = 0.881, WE (0.805), and WC (0.693).

RD scale is part of the IPIP, Emotional Intelligence 7 components, proposed by Barchard [25]. It has 10 items with dichotomous responses yes versus no. Total scores can range between 0 and 10, higher scores showing greater responsive distress. In our study, Cronbach’s Alpha for RD was 0.955.

SFD scale was retrieved from IPIP, Romanian version, and was originally developed by Gough [26]. It has 10 items with yes or no response. Higher scores mean better self-discipline. For our study, reliability of scale was moderate, Cronbach’s Alpha = 0.469.

SRGS was developed by Park et al. [31]; it has 15 items with scoring on a three-point scale, 0—disagree, 1—somewhat agree, 2—strongly agree. Scores can range from 0 to 30. The cut-off score is 28, with scores above showing individuals with relevant stress-related growth. Reliability in our sample was high, Cronbach’s Alpha = 0.855.

### 2.3. Statistical Procedures

Data collected from the online form were systematized and analyzed using IBM SPSS Statistic 20 software. Statistical procedures used for parametric data were Pearson correlation, independent sample t test, One-Way ANOVA, and for nonparametric data and not normal distributions, rho Spearman, Chi-square test, Fisher’s exact test, U Mann–Whitney test, Kruskal–Wallis test, and Generalized Linear Models [38]. Distribution normality was tested with One-Sample Kolmogorov–Smirnov test. Significance level for *p*-value was set at 0.05.

## 3. Results

### 3.1. Demographic and Health Characteristics of Participants

Our study includes 266 prehospital EM health workers. Among the participants, 67.3% are men. The age of participants ranges between 20 and 57 years, with a mean age of 38.71 years. Age distribution is normal (*p* = 0.216). Additionally, 29.7% of participants are smokers, 25.6% have an acute medical condition at the time of assessment, and 30.5% have a chronic medical condition (Table 1).

Field experience ranges from 1 to 38 years, with an average at 12.32 years, with a distribution significantly different from a standard normal distribution (*p* = 0.001). Sleep duration outside working shifts ranges from 4 to 10 h, with a mean of 7.83 h. The distribution of sleep duration differs significantly from a standard normal distribution (*p* < 0.001). Three participants report sleep durations of less than 6 h.

Women in our study tend to be older than men, with a mean age (M) of 42.25 for women, standard deviation, SD = 8.61 and M = 36.99 years for men, SD = 8.987; difference is significant at *p* < 0.001. Men and women have similar percentages of smokers, with 29.1% and 31.0%, respectively. Women (M = 14.20 years, SD = 8.59) are more experienced than men (M = 11.41 years), and this difference is significant at *p* = 0.008. However, when controlling for age, the difference in experience is significant at *p* = 0.076, indicating that the initial difference in experience can be explained by the age difference between men and women in our study.

Women exhibit a significantly higher percentage of acute medical conditions (34.5%) compared to men (21.2%), with the difference being statistically significant (*p* = 0.025.) This trend is also observed in chronic medical conditions, where women have a much higher percentage (50.6%) compared to men (20.7%), with the difference being highly significant (*p* < 0.001). Additionally, men rate their own health more positively than women, which is also statistically significant (*p* < 0.001).

### 3.2. Descriptive Statistics for Entire Sample

Participants were asked to evaluate their health status on a five-point scale, ranging from 1 (very poor) to 5 (very good). The scores ranged from 1 to 5, with an average of 4.42, and a median of 5 (SD = 0.79). The results show that most of the participants (85.7%) consider themselves to be in good and very good health (scores of 4 and 5).

Participants evaluated their work satisfaction (WS) on a ten-point scale, ranging from 1 (extremely low) to 10 (extremely high). The average score was 7.47, median = 8, SD = 1.40, scores ranging from 3 to 10. The majority of subjects (54.1%) have high levels of work satisfaction (scores of 8, 9, and 10), while only 7.1% consider their work satisfaction to be below average. No participants consider themselves as extremely and very low satisfied at work.

We requested participants to rate their subjective well-being (SWB), on a scale from 1 (extremely low) to 10 (extremely high). The average score for SWB was 9.01, median = 9.50, SD = 1.25, showing very high levels in general, with 86.5% reporting scores of 8, 9, and 10 (high SWB), and only 1.5% had scores below average (scores ≤ 5).

Participants were asked to provide information about their stress-relieving activities after working hours, which were grouped in eight broad categories. Preferences for each category are as follows: physical/sports (50.0%), intellectual (9.0%), social (3.0%), cultural/artistic (7.1%), online/PC (7.9%), house activities (7.9%), passive relaxation (9.8%), and others (5.3%).

General scores for OLBI range between 17 and 64 (Table 2), with an average of 53.41 and a median of 58, indicating that scores tend to be quite high. Although there is no established cut-off score for the Romanian population, we categorized scores into low burnout (scores ≤ 25th percentile), moderate burnout (scores between 25th and 75th percentiles) and high burnout (scores ≥ 75th percentile) [36]. The score for 25th percentile was 49.75 and for 75th percentile was 61, showing that 24.8% of the EM personnel have low burnout, 49.3% report moderate burnout, and 25.9% report high burnout. OLBI scores do not follow a normal distribution (*p* < 0.001).

Threshold scores for disengagement were 25 (25th percentile) and 29 (75th percentile). This indicates that 25.2% of the EM medical staff exhibited low disengagement, 23.3% moderate disengagement, and 51.5% high disengagement. For exhaustion, threshold scores were 25 and 32, meaning that 24.8% had low exhaustion, 46.3% had moderate exhaustion, while 28.9% showed high exhaustion.

The average scores for working excessively and working addictively (WA) are 1.96 and 2.39, respectively, both below the cut-off score of 75th percentile. In contrast, the average score for working compulsively is 2.82, which is above the cut-off score. Additionally, 39.1% of the participants show relevant scores for working excessively, 36.1% for working compulsively, and 35% for work addiction. The DUWAS scores do not follow a normal distribution (*p* < 0.001).

The average score for RD is relatively low (4.40), with scores ranging between 1 and 8. No member of the EM personnel showed high responsive distress. The average score for self-discipline is relatively high (9.63), 74.1% showing the maximum possible score, and no participant scoring below 5. The average score for stress-related growth (SRGS) is also relatively high (26.48), with 61.2% scoring above the cut-off score of 28. The distributions for RD, SFD, and SRGS scores are significantly different from a standard normal distribution, all *p*-values being below 0.001.

Men show higher levels of subjective well-being, disengagement, exhaustion, burnout, responsive distress, and self-discipline, while women show higher levels of work satisfaction, excessive and compulsive work, work addiction, and stress-related growth. All differences are significant at *p* < 0.05 (Table 3).

As shown in Table 3, acute medical conditions are relevant for exhaustion, burnout, and stress-related growth, and not relevant for disengagement, working excessively, working compulsively, work addiction, responsive distress, and self-discipline, while chronic medical conditions are relevant for disengagement, exhaustion, burnout, working excessively, responsive distress, self-discipline, and stress-related growth, and not relevant for working compulsively and work addiction.

Smoking status is not associated with any of the variables presented in Table 3.

A significant association is found between stress-recovery activities and subjective well-being (*p* = 0.022), exhaustion (*p* = 0.004), burnout (*p* = 0.021), and responsive distress (*p* = 0.033). Participants who prefer physical/sport activities and house activities tend to show higher well-being, while the lowest well-being is observed in participants who prefer social and intellectual activities. Those who prefer intellectual and social activities show lower levels of exhaustion, while the highest level of exhaustion is observed in those engaged in physical/sport and house activities. Participants engaged in cultural and other activities show lower levels of responsive distress, while the highest levels of responsive distress are observed in those who prefer intellectual and sport/physical activities.

### 3.3. Differences among Physicians, Nurses, and Paramedics (EM Personnel)

The emergency team includes a physician, a nurse, and paramedics. Therefore, we have divided the participants in three groups: physicians, nurses, and paramedics. Group distribution is 41 physicians (15.4%), 74 nurses (27.8%), and 151 paramedics (56.8%).

There is a significant difference in gender distribution, with paramedics being overwhelmingly men (98%), while nurses and physicians are mainly women, 74.3% and 70.7%, respectively. Age differs among the groups with paramedics being significantly younger than physicians and nurses. When controlling for gender, age difference between groups is significant at *p* < 0.001 (ANCOVA), with an effect size of 0.107, showing that difference in age is not only due to differences in gender distribution, with paramedics being mainly men, and nurses and physicians predominantly women, but also that women amongst paramedics are younger than women amongst nurses and physicians, and men paramedics tend to be younger than men amongst nurses and physicians (Table 4).

Physicians show the highest levels of field experience (M = 17.59 years), while paramedics have the lowest (10.24 years). When controlling for age, there is still a significant difference in field experience between groups (*p* = 0.038), although the effect size is small (eta squared = 0.025), with physicians remaining the most experienced.

Paramedics comprise a significantly lower percentage of individuals with acute and chronic medical conditions compared to nurses and physicians. The highest percentage of individuals with acute medical conditions is found in nurses, while the chronic condition rate is highest among physicians. Paramedics tend to evaluate their health status higher than nurses and physicians, although all participants report good health.

Groups differ significantly regarding their post-work stress-recovery activities (*p* = 0.001; Chi-square test) (Table 5). Paramedics show a greater percentage towards physical/sport activities (61.6%) in comparison with physicians (36.6%) and nurses (33.8%). Physicians show a greater percentage towards intellectual activities (19.5%) than nurses (12.2%) and paramedics (4.6%). Nurses have higher percentages of social, cultural, and passive relaxation activities, while physicians have higher percentages of online/PC and house activities. Results should be extrapolated with caution as 41.7% of the distribution is underrepresented (cell frequency less than 5).

To assess differences among groups for work satisfaction, subjective well-being, burnout, work addiction, responsive distress, self-discipline, and stress-related growth, we used Generalized Linear Models. This approach was chosen because the dependent variables do not show normal distributions. The models also controlled for covariates (age, sleep, experience, health status) and other factors (gender, acute and chronic medical condition, post-work stress-recovery activities) that were associated with the dependent variables.

OLBI scores per groups show that 64.9% of nurses, 36.7% of physicians, and 2% of paramedics show low burnout (scores ≤ 49), 33.7% of nurses, 43.8% of physicians, and 58.3% of paramedics show moderate levels of burnout (scores between 50 and 60), and 1.4% of nurses, 19.5% of physicians, and 39.7% of paramedics show high levels of burnout (scores ≥ 61).

Low levels of disengagement were observed in 58.1% of nurses, 26.8% of physicians, and 8.6% of paramedics. Moderate disengagement was reported by 28.4% of nurses, 22% of physicians, and 21.2% of paramedics. High disengagement was observed for 13.5% of nurses, 51.2% of physicians, and 70.2% of paramedics.

Exhaustion was reported as low by 66.2% of nurses, 36.6% of physicians, and 1.3% of paramedics, as moderate by 32.4% of nurses, 61% of physicians, and 49% of paramedics, and as high by 1.4% of nurses, 2.4% of physicians, and 49.7% of paramedics.

Work addiction was present for 33.8% of nurses, 63.4% of physicians, and 27.8% of paramedics. Rates for working excessively were 17.6%—nurses, 26.8%—physicians and 27.8%—paramedics, while for working compulsively the rates were 36.5%—nurses, 58.5%—physicians, and 29.8%—paramedics.

Most of the nurses (93.2%) and physicians (70.7%) show below moderate levels of responsive distress, but not very low, while the majority of paramedics (92.7%) show moderate levels of responsive distress. Self-discipline is quite high in all groups (scores of 9 or 10): 83.8%—nurses, 90.2%—physicians, and 98.7%—paramedics.

A total of 71.6% of nurses show SRGS scores equal to or above the cut-off score, as well as 80.5% of physicians and 50.3% of paramedics, accounting for moderate and high levels of stress-related growth among EM personnel.

There are significant differences for work satisfaction, disengagement, exhaustion, burnout, working excessively and compulsively, work addiction, responsive distress, and self-discipline (Table 6).

## 4. Discussion

Given the stressful nature of emergency medical care, we aimed to understand how EM professionals are affected by burnout, work overload, health issues, and distress they face on a daily basis, but also to identify potential emotional and behavioral coping factors like responsive distress and self-discipline. Lastly, we aimed to identify specific characteristics of each group of professionals to better understand their strengths and weaknesses.

We observed that 25.9% of the EM professionals show high burnout levels, 51.5% exhibit high levels of disengagement, and 28.9% show high levels of exhaustion. A previous study on 184 Romanian EM personnel found that 30.2% are at risk of burnout [16]. Colville et al. [10] argue for high levels of burnout amongst EM health workers, ranging from 30% to 60%, with a greater risk for physicians than nurses. Similar burnout levels, ranging between 30 and 40%, have been identified in various studies [2,8]. However, Ma et al. [39] reported significantly lower burnout levels, as low as 2.3%. Other studies, however, show higher burnout rates, ranging from 40 to 60% [9,11,12,13,14,15,40].

Work addiction among EM care professionals appears to be quite high, with 39.1% showing relevant scores for working excessively, 36.1% for working compulsively, and 35% for work addiction. Yet 54.1% of the participants report high levels of work satisfaction, which could mean that work addiction might not be all that dysfunctional for everyone with DUWAS high scores. Some might be classified as work enthusiasts, who are high in involvement but also enjoyment regarding work, but low on drive [18,41]. Assessing work addiction, Merchaoui et al. [22], report that 24% of physicians showed relevant scores and no less than 56% were at high risk for work addiction.

We observe that EM personnel exhibit moderate levels of responsive distress, indicating that while they are not overly affected by daily distress, they maintain a balance between cognitive coping and emotional sensitivity. They remain empathetic and attuned to the distress of others, including patients and their families.

Self-discipline is notably high among EM personnel, reflecting their strong adherence to procedures, which is crucial given the time-sensitive and high-responsibility nature of their work. This high level of self-discipline is likely reinforced by the military-like organizational structure of the SMURD teams in Romania, which operate under the national and regional ISU structures.

Additionally, EM personnel demonstrate a high rate of stress-related growth (61.2%), showing their capacity for professional and psychological development by finding positive outcomes from their daily stressful experiences. Identifying individual traits and organizational factors that support this growth could be key in designing effective interventions and support resources for EM medical staff.

Age correlates positively with work satisfaction, work addiction, and stress-related growth, but negatively with well-being, burnout, and responsive distress. Older EM professionals are more satisfied and experience higher work addiction but less burnout and distress. Experience is linked to lower well-being, while more sleep outside shifts correlates with burnout and self-discipline. Better self-reported health improves well-being but also associates with burnout.

Men report higher well-being and burnout, while women show more work satisfaction and stress-related growth. Acute medical conditions lower burnout, while chronic conditions increase work addiction and stress-related growth [2].

Participants who prefer physical or house activities tend to have higher well-being, while those who favor social and intellectual activities report the lowest well-being. Intellectual and social activities are linked to lower exhaustion, whereas physical and house activities correlate with higher exhaustion. Cultural activities are associated with lower responsive distress, while intellectual and sport/physical activities are linked to higher responsive distress.

Paramedics tend to be younger and mostly male, while physicians and nurses are older and predominantly female, with physicians being more experienced. Paramedics report fewer medical conditions and consider themselves healthier, with a preference for physical activities. Physicians favor intellectual and online activities, while nurses prefer social, cultural, and relaxation activities.

Burnout is highest among paramedics (39.7%) and lowest among nurses (1.4%). Paramedics also have the highest rates of disengagement (70.2%) and exhaustion (49.7%). Nurses report lower levels of disengagement, exhaustion, burnout, responsive distress, and self-discipline. Physicians exhibit higher work satisfaction, work addiction, and stress-related growth, while paramedics show higher burnout, distress, and self-discipline but lower satisfaction and work-related growth.

The higher levels of work addiction and satisfaction among physicians warrant further investigation, as they may indicate a greater enthusiasm for their work. The elevated self-discipline in paramedics could be attributed to their involvement in the ISU’s military structure. The increased burnout, disengagement, and exhaustion observed in paramedics may reflect the demands of prehospital emergency work, which involves frequent transportation, greater uncertainty, and longer shifts.

These findings suggest that while burnout and work addiction are prevalent among prehospital emergency personnel, protective factors like low to moderate responsive distress, high self-discipline, and stress-related growth are also present.

We must acknowledge certain limitations of our study: the inclusion of only 20% of Romanian counties, an imbalance in the categories of emergency medical (EM) health workers, and the varied types of activities associated with prehospital assessment for the participants (physicians and nurses: in both Emergency Departments and prehospital settings; paramedics: in prehospital settings and as firefighters).

Additionally, a cross-sectional approach does not allow us to capture the longitudinal changes over time that have been observed in other survey-based studies of EM physicians. Moving forward, a longitudinal study with a larger participant pool and additional comparative studies between prehospital emergency services, both with and without a military component, would be valuable. These studies could further compare levels of burnout, work addiction, self-discipline, responsive distress, and stress-related growth.

## 5. Conclusions

Our study shows that burnout and work addiction are present at moderate and high levels in prehospital EM medical staff. Yet EM professionals also show good levels of stress-related growth and low responsive distress, meaning that they do have good coping mechanisms even if working in a highly stressful environment.

We have found relevant differences among three categories of EM personnel: physicians, nurses, and paramedics. Physicians show higher levels of work satisfaction and work addiction. Paramedics show higher levels of burnout, responsive distress, and self-discipline, while nurses show lower levels of burnout, responsive distress, and self-discipline. The differences among physicians, nurses, and paramedics highlight the need for tailored approaches to address burnout and work addiction issues within these groups.

## Figures and Tables

**Table 1:** Demographic and health characteristics of participants.

Variable	M	SD	Median	Minimum		Maximum
Age (yrs)	38.71	9.18	40	20		57
Field experience (yrs)	12.32	8.04	13	1		38
Sleep duration outside working shifts (h)	7.83	1.12	8	4		10
Variable			frequency		percent	
Gender	men		179		67.3	
	women		87		32.7	
Smoking status	non-smokers		187		70.3	
	smokers		79		29.7	
Self-evaluation of health status	very poor		1		0.4	
	poor		4		1.5	
	average		33		12.4	
	good		71		26.7	
	very good		157		59.0	
Acute medical condition	no		198		74.4	
	yes		68		25.6	
Chronic medical condition	no		185		69.5	
	yes		81		30.5	

M—mean value of variable, SD—standard deviation.

**Table 2:** Descriptive statistics for OLBI, DUWAS, RD, SFD, SRGS (N = 266).

Variable	M	SD	Median	Minimum	Maximum
Disengagement	26.78	3.97	29	9	32
Exhaustion	26.63	6.36	29	8	32
Burnout	53.41	9.68	58.00	17	64
Working excessively (WE)	1.96	1.03	1.60	1.00	3.40
Working compulsively (WC)	2.82	0.83	2.20	1.00	4.00
Work addiction (WA)	2.39	0.90	1.90	2.00	7.40
Responsive distress	4.40	1.31	5.00	1	8
Self-discipline	9.63	0.77	10	5	10
Stress-related growth	26.48	4.80	30	13	30

OLBI—The Oldenburg Burnout Inventory, DUWAS—The Dutch Work Addiction Scale-short version, RD—Responsive Distress Scale, SFD—Self-discipline Scale, SRGS—The Stress-Related Growth Scale, N—number of participants.

**Table 3:** Differences on burnout, work addiction, responsive distress, self-discipline, and stress-related growth, regarding gender, smoking status, acute and chronic medical conditions (U Mann–Whitney test).

	Disengagement	Exhaustion	Burnout	Working Excessively	Working Compulsively	Work Addiction	Responsive Distress	Self- Discipline	Stress- Related Growth
Gender									
men (M/SD)	27.74/2.83	28.99/4.41	56.73/6.49	1.79/1.05	2.76/0.83	2.27/0.93	4.77/1.07	9.76/0.55	26.04/4.80
women (M/SD)	24.79/5.11	21.77/6.98	46.56/11.4	2.32/0.87	2.95/0.82	2.63/0.80	3.63/1.44	9.36/1.03	27.37/4.70
*p*-value	<0.001	<0.001	<0.001	<0.001	0.019	<0.001	<0.001	<0.001	0.003
Smoking status									
no (M/SD)	26.52/4.28	26.58/6.67	53.1/10.36	1.94/1.04	2.81/0.83	2.38/0.92	4.50/1.34	9.61/0.79	26.36/4.86
yes (M/SD)	27.38/3.05	26.75/5.57	54.13/7.85	2.00/1.00	2.82/0.81	2.41/0.88	4.16/1.22	9.67/0.71	26.76/4.65
*p*-value	0.490	0.600	0.927	0.349	0.914	0.415	0.057	0.468	0.725
Acute medical condition									
no (M/SD)	27.02/3.67	27.24/5.95	54.26/9.04	1.95/1.04	2.83/0.82	2.38/0.91	4.43/1.26	9.64/0.76	25.95/5.09
yes (M/SD)	26.07/4.71	24.84/7.17	50.91/11.0	2.00/0.99	2.79/0.84	2.39/0.89	4.31/1.47	9.60/0.77	28.01/3.43
*p*-value	0.145	0.003	0.020	0.423	0.673	0.673	0.330	0.666	0.019
Chronic medical condition									
no (M/SD)	27.50/2.88	28.04/5.29	55.54/7.56	1.89/1.06	2.81/0.82	2.35/0.93	4.51/1.20	9.73/0.68	25.91/5.01
yes (M/SD)	25.12/5.39	23.41/7.36	48.53/12.0	2.11/0.93	2.83/0.84	2.47/0.85	4.14/1.52	9.40/0.90	27.78/4.01
*p*-value	0.001	<0.001	<0.001	0.037	0.794	0.129	0.005	<0.001	0.001

M—mean value of variable, SD—standard deviation.

**Table 4:** Structure and differences of the 3 groups investigated: physicians, nurses, and paramedics.

Variable		EM Nurses N = 74	EM Physicians N = 41	Paramedics N = 151	Difference (*p* Value)
Gender	M (%)	19 (25.7)	12 (29.3)	148 (98)	<0.001 *
	W (%)	55 (74.3)	29 (70.7)	3 (2)	
Age (yrs)	mean	42.01	44.61	35.50	<0.001 **
	SD	9.05	8.10	8.14	
Field experience (yrs)	mean	13.65	17.59	10.24	<0.001 ***
	SD	9.11	7.86	6.66	
Smoking	yes (%)	29 (39.2)	11 (26.8)	39 (25.8)	<0.109 *
	no (%)	45 (60.8)	30 (73.2)	112 (74.2)	
Acute medical condition	yes (%)	27 (36.5)	12 (29.3)	29 (19.2)	0.017 *
	no (%)	47 (63.5)	29 (70.7)	122 (80.8)	
Chronic medical condition	yes (%)	34 (45.9)	24 (58.5)	23 (15.2)	<0.001 *
	no (%)	40 (54.1)	17 (41.5)	128 (84.8)	
Sleep (h)	mean	7.78	7.95	7.81	0.621 ***
	SD	1.11	1.22	1.10	
Self-evaluated health status	mean	4.16	4.15	4.63	<0.001 ***
	SD	0.84	0.88	0.68	

* Chi-square test; ** One-Way ANOVA; *** Kruskal–Wallis test, EM—emergency medicine, N—number, M—men, W—women, SD—standard deviation.

**Table 5:** Group distribution and differences on post-work stress-relieving activities.

Variable		EM Nurses N = 74	EM Physicians N = 41	Paramedics N = 151
Physical/Sport activities	%	33.7	36.6	61.6
Intellectual activities	%	12.2	19.5	4.6
Social activities	%	6.8	2.4	1.3
Cultural activities	%	12.2	7.3	4.6
Online activities	%	6.8	12.2	7.3
House activities	%	4.1	12.2	8.6
Passive relaxation	%	14.8	4.9	8.6
Others	%	9.4	4.9	3.4

EM—emergency medicine, N—number.

**Table 6:** Group differences for stress-related variables.

Variable		EM Nurses N = 74	EM Physicians N = 41	Paramedics N = 151	Generalized Linear Model (*p* Value)
Work satisfaction *	mean	8.14	8.59	6.83	<0.001
	SD	0.89	0.92	1.37	
Subjective well-being *	mean	8.43	8.37	9.46	0.847
	SD	1.42	1.36	0.90	
Disengagement *	mean	23.72	26.95	28.23	<0.001
	SD	4.36	4.96	2.29	
Exhaustion *	mean	20.07	24.66	30.38	<0.001
	SD	6.53	6.07	2.08	
Burnout *	mean	43.78	51.61	58.61	<0.001
	SD	10.12	10.41	3.60	
Working excessively *	mean	2.11	2.64	1.70	0.003
	SD	0.86	0.70	1.08	
Working compulsively *	mean	2.86	3.21	2.69	0.027
	SD	0.83	0.80	0.80	
Work addiction *	mean	2.49	2.93	2.17	0.008
	SD	0.82	0.69	0.93	
Responsive distress *	mean	3.27	4.17	5.01	<0.001
	SD	1.02	1.84	0.78	
Self-discipline *	mean	9.23	9.41	9.88	<0.001
	SD	1.00	0.95	0.40	
Stress-related growth *	mean	26.92	28.76	25.64	0.263
	SD	5.21	2.65	4.83	

* Distribution differs significantly from a standard normal distribution (*p*-value for One-Sample Kolmogorov–Smirnov test below 0.05), EM—emergency medicine, N—number, SD—standard deviation.

## Data Availability

The data presented in this study are available on request from the corresponding author.
